# Functional Study of the Retrotransposon-Derived Human PEG10 Protease

**DOI:** 10.3390/ijms21072424

**Published:** 2020-03-31

**Authors:** Mária Golda, János András Mótyán, Mohamed Mahdi, József Tőzsér

**Affiliations:** 1 Department of Biochemistry and Molecular Biology, Faculty of Medicine, University of Debrecen, 4032 Debrecen, Hungary; golda.maria@med.unideb.hu (M.G.); mohamed@med.unideb.hu (M.M.); 2 Doctoral School of Molecular Cell and Immune Biology, University of Debrecen, 4032 Debrecen, Hungary

**Keywords:** PEG10, paternally expressed gene 10, cell viability, cell proliferation, cis protease activity, ubiquitination, homology modeling, retroviral-like protease, protease, retrotransposon

## Abstract

Paternally expressed gene 10 (*PEG10*) is a human retrotransposon-derived imprinted gene. The mRNA of *PEG10* encodes two protein isoforms: the Gag-like protein (RF1_PEG10_) is coded by reading frame 1, while the Gag-Pol-like polyprotein (RF1/RF2_PEG10_) is coded by reading frames 1 and 2. The proteins are translated by a typical retroviral frameshift mechanism. The protease (PR) domain of RF2_PEG10_ contains an -Asp-Ser-Gly- sequence, which corresponds to the consensus -Asp-Ser/Thr-Gly- active-site motif of retroviral aspartic proteases. The function of the aspartic protease domain of RF2_PEG10_ remains unclear. To elucidate the function of PEG10 protease (PR_PEG10_), we designed a frameshift mutant (_fs_RF1/RF2_PEG10_) for comparison with the RF1/RF2_PEG10_ form. To study the effects of PR_PEG10_ on cellular proliferation and viability, mammalian HEK293T and HaCaT cells were transfected with plasmids coding for either RF1/RF2_PEG10_, the frameshift mutant (_fs_RF1/RF2_PEG10_), or a PR active-site (D370A) mutant _fs_RF1/RF2_PEG10_. Our results indicate that _fs_RF1/RF2_PEG10_ overexpression results in increased cellular proliferation. Remarkably, transfection with _fs_RF1/RF2_PEG10_ had a detrimental effect on cell viability. We hypothesize that PR_PEG10_ plays an important role in the function of this retroviral remnant, mediating the proliferation of cells and possibly implicating it in the inhibition of apoptosis.

## 1. Introduction

**Figure 1:**
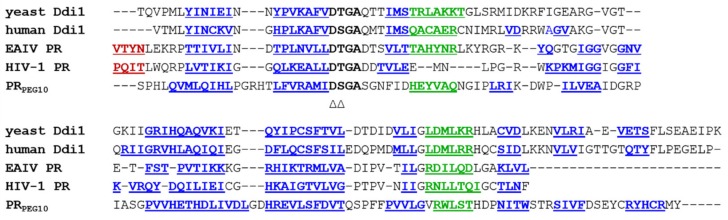
Structure-based sequence alignment of PR_PEG10_ with some retroviral and retroviral-like proteases. Secondary structures of yeast (2I1A.pdb) and human Ddi1 (3S8I.pdb), EAIV (1FMB.pdb), and HIV-1 (5HVP.pdb) PRs are indicated based on crystal structures, while the predicted secondary structural arrangement of PR_PEG10_ is shown based on in silico prediction. D-S/T-G-A active site motif residues are indicated by bold letters, the secondary structural elements are bold and underlined, while α-helices are colored in green. The color code of β-sheets in this figure corresponds to that of Figure 2—the N-terminal β-strands of HIV-1 and EIAV PRs, (which are involved in the formation of dimer interface) are red, while all other β-strands are blue. Triangles show PR_PEG10_ residues modified in this study to design D370A-_fs_RF1/RF2_PEG10_, D369A-RF1/RF2_PEG10_, and S371A-_fs_RF1/RF2_PEG10_ mutant proteins.

Paternally expressed gene 10 (*PEG10*) is an imprinted gene located on the human chromosome 7q21 and is known to have evolved from a retroviral element, although it has lost the ability to replicate independently [1]. This domesticated retroviral remnant has been shown to be essential for embryonic development in mice; previous studies demonstrated that mutations in the coding sequence of the gene are lethal in the embryonic stage because of defects in placental development [2]. Additionally, upregulated *PEG10* expression has been observed in malignancies such as hepatocellular carcinoma [3,4,5,6], embryonic kidney Wilms’ tumor [7], lung cancer [8], breast cancer [9], and pancreatic cancer [10,11]. Although endogenous expression of *PEG10* had previously been detected using antibodies in the human liver cancer cell line HepG2 and human embryonic kidney cells (HEK), more sensitive methods revealed that other cell lines, such as breast tumor and large-cell lung carcinoma cells, also express *PEG10* [12]. The mRNA of *PEG10* encodes at least two protein isoforms: the major PEG10 protein product reading frame 1 (RF1_PEG10_) and reading frames 1 and 2 (RF1/RF2_PEG10_), which are translated by a typical retroviral-1 ribosomal frameshift mechanism [13,14,15,16]. RF1_PEG10_ encodes a Gag-like protein, and RF1/RF2_PEG10_ encodes a Gag-Pol-like polyprotein that is characteristic to retroviruses and retroelements. Additionally, RF1_PEG10_ has a conserved major homology domain and a CCHC-type Zn-finger motif in its structure (Cys-X_2_-Cys-X_4_-His-X_4_-Cys). Shortly following the -1 ribosomal frameshift site, RF2_PEG10_ contains a consensus -Asp-Ser-Gly- sequence motif (Figure 1) that is characteristic of retroviral aspartic proteases; this domain is followed by a truncated reverse transcriptase domain [17].

While the anti-apoptotic role of RF1_PEG10_ was previously investigated [18,19,20], the role of RF2_PEG10_ has not yet been elucidated. The predicted post-translational modifications of RF2_PEG10_, such as phosphorylation and dephosphorylation, may be essential for the regulation of PEG10 function [15]. Considering its importance, the −1 translational frameshift mechanism of RF1/RF2_PEG10_ has been thoroughly investigated. Lux et al. [12] cloned the entire RF1/RF2_PEG10_ sequence into an eukaryotic expression vector. RF1/RF2_PEG10_ was then expressed in transfected COS-1 cells, and the cell lysate was analyzed by Western blot. Three protein bands were identified that corresponded to the full length of RF1/RF2_PEG10_, as well as RF1_PEG10_ and a shorter N-terminal fragment (PEG10-cleaved N-terminus fragment), which was considered to be most likely a cleavage product of an aspartic protease [12]. Analysis of the proteolytic activity of PEG10 protease (PR_PEG10_) was previously attempted using an active-site mutant, where the conserved aspartate was mutated to alanine, thereby impairing its catalytic activity [15]. Even though the authors verified the proteolytic activity of PR_PEG10_, it is unclear whether this proteolytic activity is required for the function of PEG10, or whether PR_PEG10_ has simply persisted as an evolutionary remnant. We assumed that the PEG10 is involved in the regulation of cell proliferation and has anti-apoptotic effects due to the protease (PR) activity of RF2_PEG10_. Based on this hypothesis, and if proven correct, PR_PEG10_ may be effectively targeted for chemotherapeutic purposes.

In this study, we focused on the aspartic protease domain of RF1/RF2_PEG10,_ a part of RF2_PEG10_. Although PR_PEG10_ is believed to mediate polyprotein processing in a similar manner to other retroviral PRs [15], neither the biochemical characteristics of this protease nor its functional importance have been established to date. Therefore, we aimed to investigate the activity of PR_PEG10_ and study its involvement in the function of PEG10. The activity of PR_PEG10_ was proven by investigating autoproteolysis of wild-type and active site mutant RF1/RF2_PEG10_ proteins in the lysates of transfected HEK293T cells. Furthermore, functional studies were performed in order to investigate whether overexpression of frameshift mutant _fs_RF1/RF2_PEG10_ affects the proliferation and viability of transfected human embryonic kidney 293T (HEK293T) and human keratinocyte HaCaT cells.

## 2. Results

### 2.1. In Silico Analyses

Data about PR_PEG10_ are limited, therefore in silico methods were applied to investigate its structural characteristics. Results of secondary structure prediction implied that the conserved active-site motif and the overall secondary structure element arrangement of PR_PEG10_ are shared with retroviral PRs (Figure 1). Disorder prediction revealed that the region in close proximity to the frameshift site (247–348) is unstructured. A region located near the catalytic D-S-G-A motif was predicted to adopt an α-helical conformation (Figure 1). This helical region presumably corresponds to an additional helical insert that is not present in most retroviral PRs but is present in equine infectious anemia virus (EIAV) protease [21], as well as in both human and yeast DNA-damage-inducible 1 (Ddi1) proteins [22]. The C-terminal region of the protease domain was predicted to contain three β-sheets connected by short loops (Figure 1), implying that the homodimeric PR_PEG10_ has a six-stranded dimer interface.

**Figure 2:**
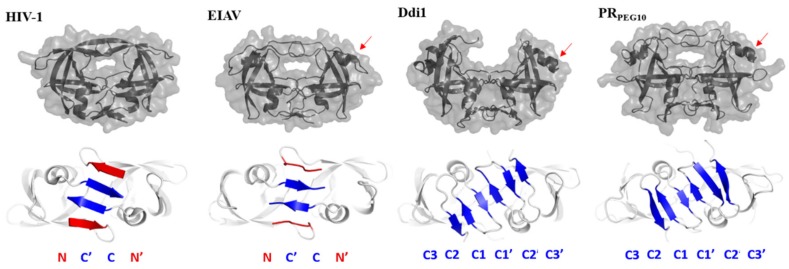
The predicted folding pattern of PRPEG10 is similar to that of other homodimeric aspartic proteases. Overall structures represented here are based on crystal structures of HIV-1 PR (PDBID: 7HVP), EIAV PR (PDBID: 1FMB), and human Ddi1 protease (PDBID: 3S8I), while the proposed model structure of PR_PEG10_ is shown. Red arrows indicate additional helical inserts in the front views of PR structures (upper panel). Dimer interfaces of the PRs are enlarged in the bottom views of PR structures (lower panel); only the N- and C-terminal β-sheets are colored (red and blue, respectively).

A putative structure of homodimeric PR_PEG10_ was proposed by homology modeling in this study (Figure 2). The N- and C-terminal boundaries of PR_PEG10_ have not yet been experimentally determined; hence, the proposed termini of the protease domain are only approximate. The region upstream of the putative N-terminal residue (Ser346) and the proximity of the frameshift site (Gly319 and Lys320 residues) were also predicted to be unstructured, which implied that these regions do not belong to the globular protease fold. The predicted presence of three β-sheets at the C-terminal end of PR_PEG10_ implies similarity to the dimer interfaces of Ddi1 proteins; therefore, their structures were used to build a six-stranded dimer interface for homodimeric PR_PEG10_. Additionally, Ddi1 proteins were found to be useful templates due to the presence of an additional helical insert, which is also present in EIAV PR [21]. The crystal structure of EIAV PR was used as a template to model closed conformational flap regions of PR_PEG10_, since the flaps of Ddi1 proteins are disordered in electron density maps [22] (Figure 2). However, template structures showed <20% sequence identity to PR_PEG10_; despite this, these proteins share high structural similarity [22].

Based on the results of secondary structure prediction and homology modeling, PR_PEG10_ shares its overall fold with retroviral PRs (Figure 2). The dimer interface of PR_PEG10_ was predicted to resemble those of Ddi1 proteins, and consists of only C-terminal β-sheets showing no alternation. In contrast, both the N- and C-terminal β-sheets are involved in the formation of the HIV-1 PR dimer interface, showing interdigitation (Figure 2). A putative C-terminal residue of the protease domain was predicted to be located near the third β-sheet, and the dimer interface was predicted to be not followed by a longer C-terminal extension, since no secondary structural elements were predicted for this region. The proposed model structure may be used to support the biochemical characterization of the protease, including mutation design and computational calculations.

To inactivate PR_PEG10_, we mutated the catalytic aspartate to alanine to create a D370A mutant protein. Mutation of Thr to Ala in the D-T-G-A active-site motif of HIV-1 PR was previously reported to result in a virtually inactive enzyme, since the dimerization of monomers is inhibited due to the lack of the key “fireman’s grip” interactions [23]. To investigate whether PR_PEG10_ dimerization is similar to that of HIV-1 PR and to correlate the findings of in vitro experiments to the homology model, an S371A-_fs_RF1/RF2_PEG10_ mutant was also designed, in which the D-S-G-A catalytic motif’s Ser residue was mutated to Ala.

**Figure 3:**
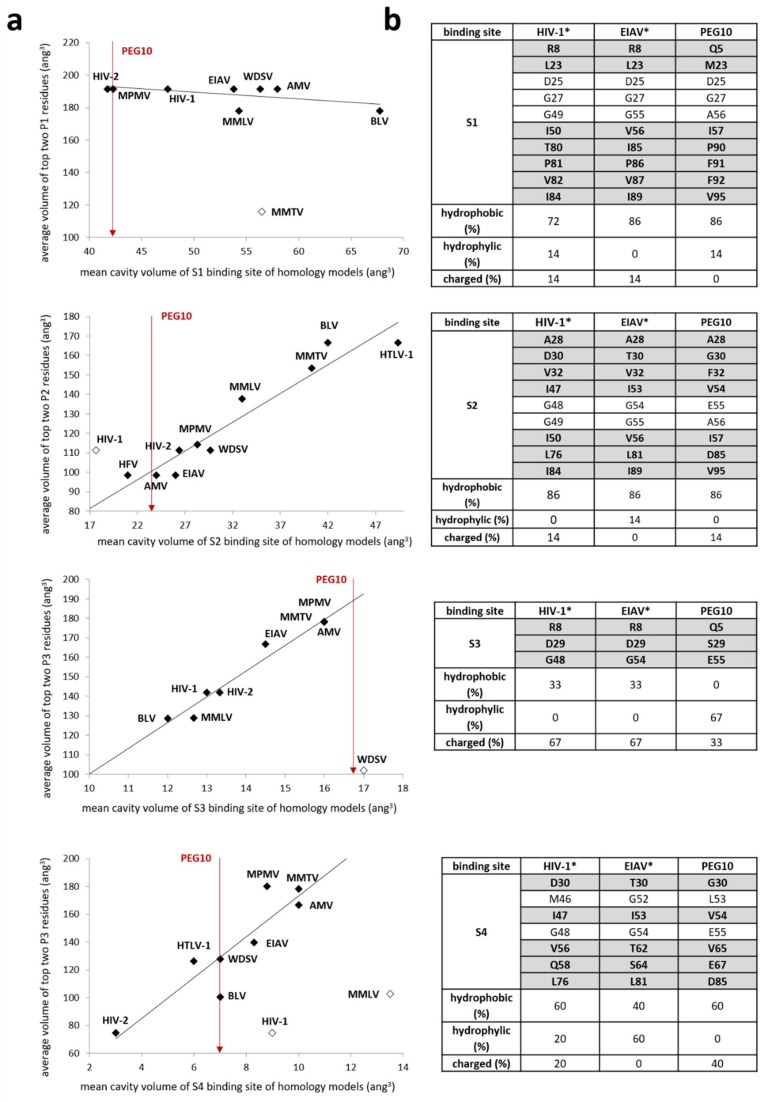
Comparison of mean cavity volumes and substrate binding site compositions. Mean cavity volumes (left graphs) and amino acid compositions (right tables) of substrate binding sites are shown for HIV-1, EIAV, and PEG10 proteases. Graphs represent the average volumes of most preferred residues plotted against the mean cavity volumes of the substrate binding sites. The most preferred residues and cavity volumes have been determined previously for retroviral proteases in vitro and in silico, respectively [24,25]. Black diamond symbols indicate values used for correlation analysis, while opened diamonds show values that have been excluded from the correlation. Only mean cavity volumes are indicated for PR_PEG10_ binding sites (shown by red arrows), since the volumes of preferred residues have not been determined experimentally to date (**a**). Substrate binding site compositions of HIV-1 and EIAV PRs (*) were determined in previous studies [24,25] and structural alignment based on homology model structure was used to identify residues in the case of PR_PEG10_. Bold letters indicate that side-chain–side-chain interaction may occur. Residues were classified as follows: hydrophobic: A, V, I, L, F, W, C, M, P, and G; hydrophilic: S, T, N, Q, Y, and H; charged: D, E, K, and R (**b**).

Beside comparing the overall folding and secondary structural arrangement of PR_PEG10_ to that of retroviral or other retroviral-like proteases, we used the model structure to investigate the substrate binding sites of the enzyme. In our previous comparative studies, the mean cavity volumes of S1–S4 substrate binding sites were calculated to determine the P1–P4 specificities of retroviral PRs, respectively [24,25]. Although in vitro specificity studies have not yet been performed for PR_PEG10_, results of in silico calculations can be used to compare the compositions of substrate binding sites to those of other PRs (Figure 3a). The volume of the S1 site of PR_PEG10_, which is composed of mainly hydrophobic residues, was determined to be similar to that of HIV-2 and Mason–Pfizer monkey virus (MPMV) PRs, and smaller than that of HIV-1 and EIAV PRs. However, retroviral PRs may show remarkable differences in the size of the S1 cavity and they were found to have similar preferences for Phe and Tyr residues in the P1 position [25]. In accordance with this, PR_PEG10_ may also potentially prefer to bind medium- or large-sized hydrophobic residues at the S1 site. The cavity volume of the S2 site of PR_PEG10_ resembles that of a group of proteases that exhibit preference for Ala and Cys at this position [25]. Similar to that of HIV-1 and EIAV PRs, the S2 cavity of PR_PEG10_ also consists of mainly hydrophobic residues. The S3 site of PR_PEG10_ was calculated to be large and contains exclusively hydrophilic residues, which implies a preference for polar P3 residues at this site. This is similar to the S3 sites of HIV-1 and EIAV PRs, which do not discriminate P3 substrate residues based on hydrophobicity [25]. Although the S4 pocket is less defined than the others due to its close proximity to the protein surface, its size was also calculated. PR_PEG10_ was found to have an average-sized S4 site, with a similar ratio of hydrophobic and hydrophilic residues to that of HIV-1 PR (Figure 3b). Results were used for the estimation of an autoproteolytic cleavage site, which is discussed below.

### 2.2. Proteolytic Activity of PR_PEG10_

**Figure 4:**
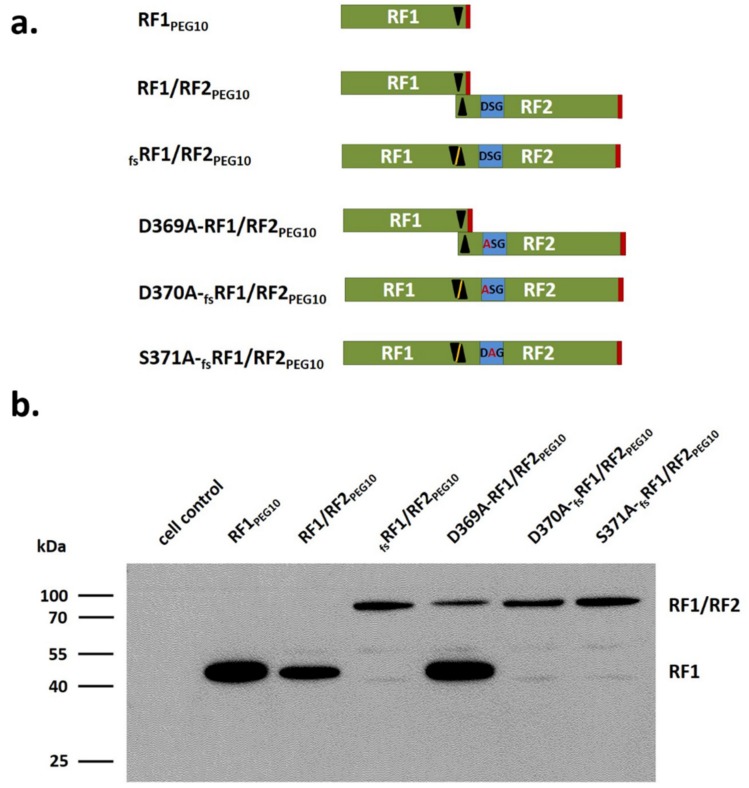
Comparison of the studied forms of PEG10. Schematic structures of the PEG10 proteins designed in the present study. RF1 and RF2 are colored by green, the black triangles indicate the frameshift site, and red markings show the termination codons of the translation, while blue color highlights active site motif (**a**). Expression of different forms of RF1/RF2_PEG10_ in HEK293T cells was detected with anti-PEG10 antibody (**b**).

For the characterization of PR_PEG10_, a total of six protein variants were prepared (Figure 4a). In addition to RF1/RF2_PEG10_ and its frameshift mutant form (_fs_RF1/RF2_PEG10_), active site protease mutants were also studied (Figure 4a).

All proteins were expressed in HEK293T cells, which were transfected by equal amounts of plasmid DNAs for each variant, while expression of the different PEG10 proteins was detected by Western blot (Figure 4b). Since the anti-PEG10 used for Western blot only targeted RF1_PEG10_ (1–325 res), we could not detect RF2_PEG10_ alone.

HEK293T cells have a low basal level of endogenous PEG10 expression according to the Human Protein Atlas database (https://www.proteinatlas.org). In agreement with this, the protein was not detected in HEK293T cells by Western blot; therefore, non-transfected cells were used as control. In the lysates of cells transfected with RF1/RF2_PEG10_ plasmid, only RF1_PEG10_ was detectable, which indicated the previously described low frameshift efficiency [16] and proteolytic processing of the RF1/RF2_PEG10_ protein. For the D369A-RF1/RF2_PEG10_ mutant, the RF1/RF2_PEG10_ protein was also detectable, which implied that mutation of the protease’s active site prevented autoproteolysis (Figure 4b).

**Figure 5:**
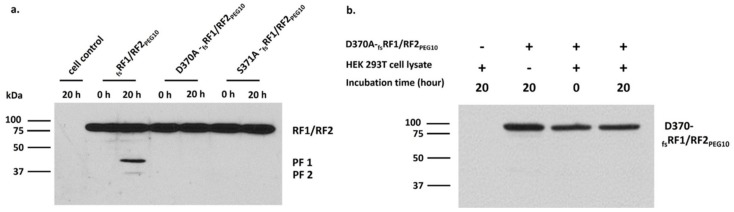
The appearance of cleavage fragments implies proteolytic activity of RF1/RF2_PEG10_. Lysates of cells transfected with _fs_RF1/RF2_PEG10_ or with active-site mutant _fs_RF1/RF2_PEG10_ were incubated at 37 °C and then analyzed by Western blot. Upper bands indicate _fs_RF1/RF2_PEG10_ proteins, and lower bands indicate proteolytic fragments (PF1: ~45 kDa; PF2: ~37 kDa). Results are representative of three independent experiments (**a**). The lysate of cells overexpressing D370A-_fs_RF1/RF2_PEG10_ was incubated with the total cell lysate of non-transfected HEK293T cells (**b**). “+” denotes the presence, while “–“ denotes the absence of the given component.

To ensure overexpression of RF1/RF2_PEG10_ and explore the effects of active-site mutations on autoproteolysis, a frameshift variant (_fs_RF1/RF2_PEG10_) and active site mutants were also designed (D370A- and S371A-_fs_RF1/RF2_PEG10_) (Figure 5a). Following incubation of the lysate of cells overexpressing _fs_RF1/RF2_PEG10_, we observed the appearance of bands at ~45 kDa and ~37 kDa (Figure 5a). These bands were thought to correspond to those processing fragments (PF1: ~45kDa; PF2: ~37kDa) that resulted from the proteolytic activity of _fs_RF1/RF2_PEG10_ [15]. The autoproteolytic fragments were not detected for the catalytic aspartate mutant (D370A-_fs_RF1/RF2_PEG10_) protein, indicating that active site mutation abolished its proteolytic activity, supporting the hypothesis that PR_PEG10_ has maintained its ability for autoproteolysis during its evolution. Similar to the D370A mutation, the S371A mutation also resulted in the inactivation of PR_PEG10_ (Figure 5a).

To exclude the possibility that the _fs_RF1/RF2_PEG10_ protein was processed in our experiments by cellular proteases of HEK293T cells, the lysate of cells overexpressing the D370A catalytic mutant protein was incubated with the lysate of non-transfected HEK293T cells. In this case, no proteolytic fragments were detected by Western blot, even after a long incubation time. This proved that PR_PEG10_ was responsible for the observed processing of _fs_RF1/RF2_PEG10_ protein rather than cellular proteases (Figure 5b).

In the experiments shown in Figure 5, a long incubation time (20 h) was applied to provide sufficient processing of the RF1/RF2 precursors, to prove the success of protease inactivation, and to ensure that active site mutants do not undergo self-proteolysis. In consideration, we applied a long incubation time for the samples in other experiments as well; however, we observed autoproteolysis even with a shorter incubation time (see later in Section 2.5).

### 2.3. Estimation of an Autoproteolytic Cleavage Site

Computational calculations based on homology model structures may aid specificity studies [24,25], and together with the results of in vitro experiments can be used to predict possible cleavage site sequences. HIV-1 PR is the most studied retroviral protease, and several methods are available to predict its cleavage sites within target sequences; however, such predictions may be more problematic or less accurate for less-studied enzymes. While the autoproteolytic recognition sequences of PR_PEG10_ are unknown and experimental specificity studies have not been yet performed, we used the proposed model structure to predict an autoproteolytic cleavage site within RF1/RF2_PEG10_.

**Figure 6:**
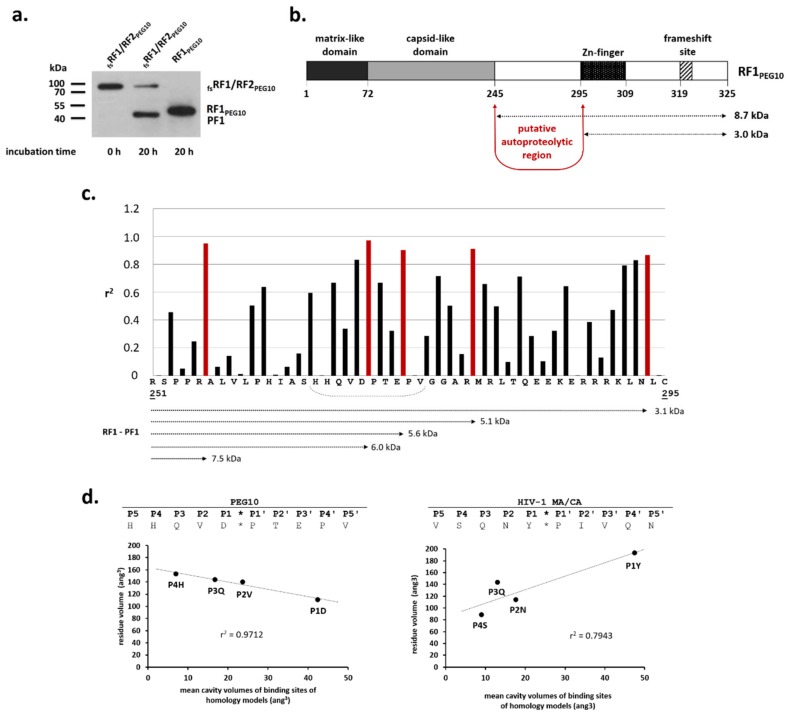
Estimation of the autoproteolytic region in _fs_RF1/RF2_PEG10_. To identify the region in _fs_RF1/RF2_PEG10_ where the autoproteolysis may occur, the molecular weight of PF1 was compared to that of RF1_PEG10_. A representative blot image of three independent experiments is shown (**a**). Approximately 4–5 kDa difference in the molecular weight indicated that RF1_PEG10_ may be cleaved prior to the frameshift site, between the C-terminal capsid-like domain and the Zn-finger motif (**b**). The sequence of RF1_PEG10_ was analyzed in order to identify a potential cleavage site in the region between the capsid-like domain and the Zn-finger motif. The volumes of P4–P1 residues were correlated with the calculated cavity volumes of S4–S1 sites, respectively (see cavity volumes in Figure 3). Each arrow shows a putative cleavage position for which a correlation coefficient (r2) has been determined. The highest values are shown in red. The calculated difference between RF1_PEG10_ and PF1 is also shown for some cleavage positions. The dashed line shows the sequence for which the correlation analysis is shown in the graph (**c**). For PEG10, the graph shows the correlation between volumes of P4–P1 residues and S4–S1 cavities for the 266HQVD*PTEP273 cleavage site. For comparison, calculation was performed for the HIV-1 MA/CA cleavage site (SQNY*PIVQ) using the cavity volumes published previously (**d**) [24,25].

The difference between the molecular weight of the PF1 proteolytic fragment and RF1_PEG10_ protein was estimated by analysis of Western blot images (Figure 6a), and was found to be between 4 and 5 kDa. This difference implied the occurrence of a cleavage site between the capsid-like domain and the Zn-finger motif of RF1_PEG10_ (Figure 6b). In order to predict a possible cleavage site, volumes of P4–P1 residues were correlated with the calculated cavity volumes of S4–S1 sites, respectively (see cavity volumes in Figure 3). Correlation analysis using a 4-residue window (Figure 6c) revealed the highest value for the HHQVD*PTEPV sequence (Figure 6d), which may correspond to a potential cleavage site due to the following reasons: (i) the highest correlation was observed for this cleavage site if volumes of P4–P1 residues were correlated with those of S4–S1 cavities, respectively; (ii) Gln and Glu residues can be found in P3 and P3’ positions, respectively, which is in agreement with the hydrophilic residue composition of the S3 substrate binding cavity; and (iii) despite containing an unusual residue (Asp) in the P1 position, the proposed sequence resembles the type 1 retroviral protease cleavage sites [26], which also contain Pro at the site of cleavage (in the P1′ position).

### 2.4. Ubiquitination of PEG10

**Figure 7:**
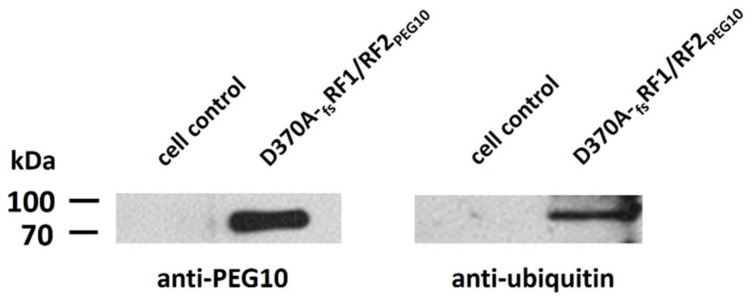
Western blot analysis revealed ubiquitination of PEG10. To evaluate whether the PEG10 protein undergoes ubiquitination, D370A-_fs_RF1/RF2_PEG10_ was purified and immunoblotted using anti-PEG10 and anti-ubiquitin primary antibodies. Total cell lysates of HEK293T cells were used as control and underwent the same technical procedures as D370A-_fs_RF1/RF2_PEG10_ (cell lysis, Ni-chelate affinity chromatography).

Ubiquitination of D370A-_fs_RF1/RF2_PEG10_ was detected by Western blot using anti-ubiquitin antibody (Figure 7), proving that the protein undergoes ubiquitination. The molecular weight of the protein was estimated in relation to molecular weight markers after SDS-PAGE, and the obtained difference implied di-ubiquitination of the protein (the molecular weight of ubiquitin is ~8.5 kDa).

In addition, we suppose that the ~8 kDa difference observed between the molecular weights of the PF1 and PF2 product bands (Figure 5a) may be the result of ubiquitination; accordingly, the ~45 kDa cleavage fragment may correspond the ubiquitinated form of the ~37 kDa product. The ~37 kDa product containing the intact N-terminal region of RF1 had relatively lower band intensity compared to the other band (~45 kDa), both in the previous report [15] and the herein described blot image. To prove this, the eukaryotic expression system needs to be applied for protein expression, because *Escherichia coli* cells lack an ubiquitination system. Moreover, proteolytic fragments may be hardly detectable in total cell lysates, where a significant amount of ubiquitinated cellular protein may also be detected.

### 2.5. Trans Activity of PR_PEG10_

**Figure 8:**
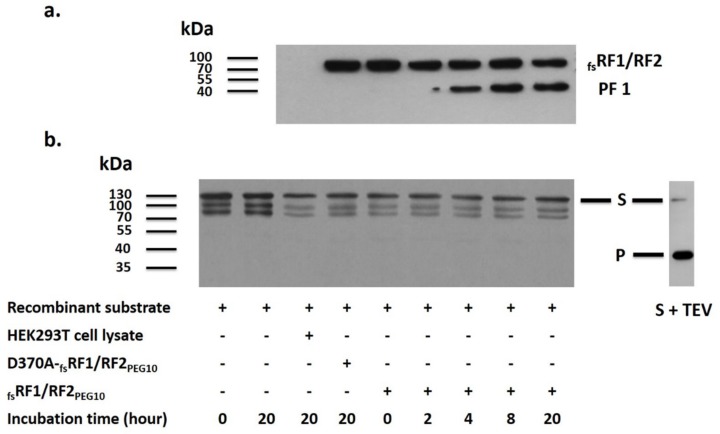
PR_PEG10_ does not exhibit trans activity. The lysate of _fs_RF1/RF2_PEG10_-transfected cells was dialyzed against storage buffer and later incubated with a recombinant protein substrate (**S**) containing potential autolytic sites. For Western blot, anti-PEG10 antibody was applied to follow the autoproteolysis of _fs_RF1/RF2_PEG10_. Sample compositions are shown at the bottom of the figure; samples shown in figure part A did not contain recombinant protein substrate (**a**). Furthermore, anti-MBP antiserum was used to follow changes in the full-length recombinant protein. The recombinant substrate was digested with TEV PR (S+TEV) as well. The product is indicated with the letter P (**b**). Results are representative of three independent experiments. “+” denotes the presence, while “–“ denotes the absence of the given component.

To test the catalytic activity of PR_PEG10_, we designed a His_6_-MBP-_fs_RF1/RF2_PEG10_-mTurquoise2 protein substrate containing the 1–345 residues of the full-length human _fs_RF1/RF2_PEG10_ protein (MBP denotes maltose binding protein). This region was believed to contain putative autoproteolytic site(s) of PR_PEG10_ (see Figure 6b). The His_6_-MBP-f_s_RF1/RF2_PEG10_-mTurquoise2 protein was expressed in BL21(DE3) *Escherichia coli* cells, and after its purification with nickel-nitrilotriacetic acid (Ni-NTA) magnetic agarose beads, the purified recombinant protein was applied as a substrate for PR_PEG10_. Even in the presence of multiple possible cleavage sites in the recombinant protein, cleavage reactions revealed no proteolysis of the substrate, which implied the lack of trans activity of the protease (Figure 8b). In parallel with the cleavage reactions, autoproteolysis was also studied at all time points, and the results indicated autoproteolytic activity (cis activity) of PR_PEG10_ (Figure 8a). A control cleavage reaction was also performed by TEV PR, and the effective cleavage indicated proper folding of the recombinant substrate (Figure 8b). Based on these results, we suppose that autoproteolysis of the RF1/RF2_PEG10_ protein may block the trans activity of PR_PEG10_.

To investigate the protease activity of the recombinant protein, _fs_RF1/RF2_PEG10_ was expressed in a bacterial expression system as well, but we failed to detect proteolytic activity of the purified protein.

### 2.6. Effect of pH on Autoprocessing

**Figure 9:**
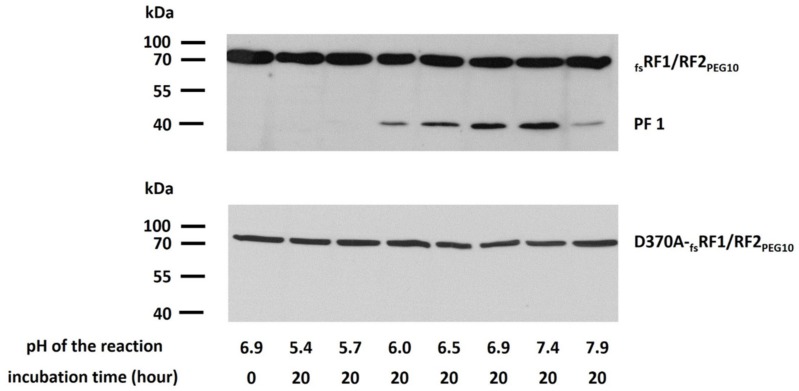
Processing of _fs_RF1/RF2_PEG10_ is dependent on pH. Autoprocessing of _fs_RF1/RF2_PEG10_ (**a**) and D370A-_fs_RF1/RF2_PEG10_ (**b**) was studied in META buffer (pH range: 5.4–7.9). Release of PF1 autoproteolytic fragment was not observed for the protease mutant (lower blot image). Results are representative of two independent experiments.

The dependence of _fs_RF1/RF2_PEG10_ autoproteolytic activity on pH was also studied in the pH range of 5–8 (Figure 9). The appearance of the PF1 proteolytic cleavage product was followed by detection of _fs_RF1/RF2_PEG10_ using Western blot. We observed increasing band intensities at ≥ 6.0 pH, while lower pH was found to be suboptimal for PR_PEG10_. The optimal pH for the autoprocessing of _fs_RF1/RF2_PEG10_ was close to neutral (between 6.9 and 7.4) (Figure 9a). The D370A mutant _fs_RF1/RF2_PEG10_ was used as control, and as expected, it exhibited no autoproteolytic activity (Figure 9b).

### 2.7. Effects of Protease Inhibitors on Autoprocessing

**Figure 10:**
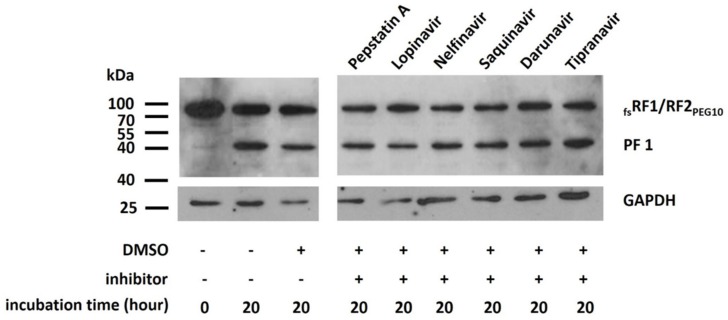
PR_PEG10_ autoproteolysis is not inhibited by the tested inhibitors. Autoproteolysis of _fs_RF1/RF2_PEG10_ was studied in vitro in the presence of the PR inhibitors using pepsatin A, lopinavir, nelfinavir, saquinavir, darunavir, and tipranavir. Each inhibitor was dissolved in DMSO and applied at a 10 µM final concentration. Results were obtained by Western blot using anti-PEG10 antibody. Results are representative of two independent experiments. “+” denotes the presence, while “–“ denotes the absence of the given component.

Using in vitro experiments, we assessed whether pepstatin A, lopinavir, nelfinavir, saquinavir, darunavir, and tipranavir PR inhibitors are able to inhibit PR_PEG10_ self-processing. The control sample contained only DMSO, since the inhibitors were dissolved in this organic solvent. None of the tested inhibitors showed significant inhibition of the protease activity; the formation of PF1 product was comparable to that in the control sample (Figure 10).

### 2.8. Effect on Cellular Proliferation and Viability

Involvement of PEG10 in carcinogenesis in multiple cell lines had already been reported [3,4,5,6,7,8,9,10,11]; therefore, after investigating the characteristics of PEG10 autoproteolysis, the potential role of PR_PEG10_ in cellular proliferation and the effect of its overexpression on cell viability were also studied.

**Figure 11:**
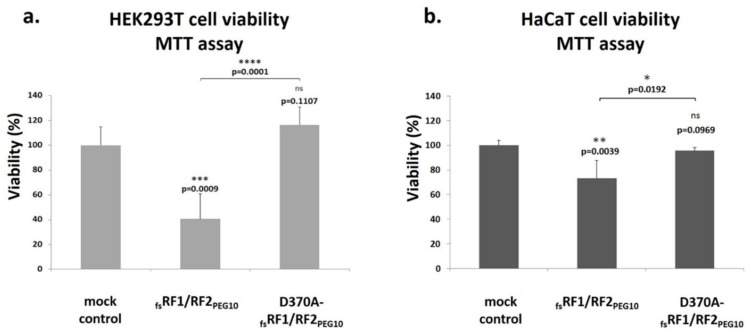
_fs_RF1/RF2_PEG10_ decreases the viability of HEK293T and HaCaT cells. The role of PR_PEG10_ in the cell viability of HEK293T (**a**) and HaCaT cells (**b**) was investigated using the MTT assay, which was performed 1 day after transfection. Mock control cells were transfected with pQE-TriSystem plasmid. Significance values were determined as compared to mock control, otherwise they are indicated. Note: ****p ≤ 0.0001; ***p  ≤  0.001; **p  ≤  0.01; *p ≤ 0.05; ns = non-significant (*p* > 0.05). Error bars represent SD (*n* ≥ 4).

Overexpression of _fs_RF1/RF2_PEG10_ was found to significantly decrease the viability of HEK293T and HaCaT cells maintained in optimal growth conditions compared to the mock control. In the case of the inactive protease mutant D370A-_fs_RF1/RF2_PEG10_, no significant change in cell viability was observed compared to the mock control (Figure 11).

**Figure 12:**
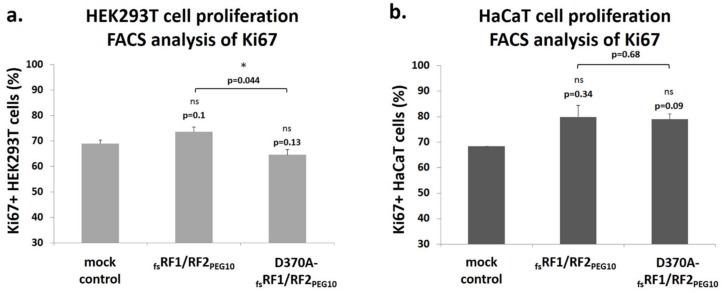
PR_PEG10_ influences the proliferation of HEK293T cells. In the cell proliferation assay, HEK293T (**a**) and HaCaT cells (**b**) were stained with FITC-conjugated anti-Ki67 antibody, which was detected using a flow cytometer (FACS). Significance values were determined as compared to mock control, otherwise they are indicated. Note: *p  ≤  0.05; ns = non-significant (*p* > 0.05). Error bars represent SD (*n* ≥ 2).

In agreement with the results of previous studies, we found that overexpression of PEG10 did not result in remarkable promotion of cell growth in HEK293T cells [4]. However, when we compared proliferation of cells overexpressing _fs_RF1/RF2_PEG10_ or D370A-_fs_RF1/RF2_PEG10_, a significant difference was observed. In the case of _fs_RF1/RF2_PEG10_, the percentage of Ki67-positive HEK293T cells was significantly higher compared to those expressing the inactive protease mutant, suggesting that PR_PEG10_ plays an active role in the promotion of proliferation. We also investigated the rate of cell proliferation in HaCaT cells; however, as these cells undergo rapid division (doubling time of 24 h), no significant difference was observed (Figure 12).

The characteristics of the protease mutant PR_PEG10_ had not been previously investigated in detail. Studies that revealed the oncogenic potential of *PEG10* focused on RF1_PEG10_, while RF2_PEG10_ remained understudied. Based on our results, we can conclude that both RF1_PEG10_ and RF2_PEG10_ play significant roles in cell viability and proliferation in HEK293T and HaCaT cells. We hypothesize that RF1/RF2_PEG10_ may neutralize the oncogenic potential of RF1_PEG10_ by a self-inactivation mechanism.

## 3. Discussion

In this study, we investigated the structural and biochemical characteristics of the aspartic protease domain encoded by the human retrotransposon-derived gene *PEG10*.

The three-dimensional structure of PR_PEG10_ has not been solved experimentally to date; therefore, in silico prediction algorithms were applied to estimate structural characteristics of the protease structure. Based on the results of secondary structure prediction and homology modeling, PR_PEG10_ shares its overall fold with those of retroviral PRs (Figure 2). PR_PEG10_ was found to show the highest sequence and structural similarity with DNA-damage-inducible 2 (Ddi2) and Ddi1 PRs [22,27,28]; each of these retroviral-like PRs contains an additional helical insert and has a six-stranded dimer interface organization, which was not found to be characteristic of retroviral PRs [29]. In addition, the D-S-G-A active site motif of human PR_PEG10_ is also identical to that of human Ddi1 and Ddi2 PRs, but this consensus motif is followed by a Ser residue (D-S-G-A-S) in PR_PEG10_ (Figure 1). Ser in this position is not characteristic of retroviral or Ddi1/Ddi2 PRs [29] but can be found in the Ty1 retrotransposon PR [30]. The D-T/S-G-A active site motif of most retroviral PRs is followed by an Asp residue, but in contrast with this Asp residue, a Ser in this position does not enable salt-bridge formation between PR_PEG10_ subunits. In the sequence motif of the consensus helix, retroviral PRs (excepting spumaretrovirus PRs) contain the G-R-N motif, but similarly to Ddi1 and Ddi2 proteins, PR_PEG10_ also contains a different sequence (G-V-R) in this helix, and thus lacks the corresponding interactions that are provided by the Arg residue of the G-R-N motif [29]. Structural analysis of retroviral and Ddi1 and Ddi2 PRs revealed correlation between the dimer interface organization and the intermonomeric contacts [29]. No data are available for in vitro dimer stability of PR_PEG10_, but high structural similarity implies that its dimer stability may be comparable with those of Ddi1/Ddi2 PRs and may be lower than that of HIV-1 PR.

To investigate the biochemical characteristics of PR_PEG10_ in vitro, we expressed different forms of PEG10 in HEK293T mammalian cell line (Figure 4a), and detected these in total cell lysates by Western blot analysis (Figure 4b). We observed two proteolytic fragments (PF1: ~ 45 kDa and PF2: ~ 37kDa); the ~37 kDa product was previously found to contain the intact N-terminus of the protein and is part of RF1 [15]. Proteolytic fragments did not appear when *PEG10* harboring an active-site mutation of PR_PEG10_ was expressed (Figure 5). As observed in the case of HIV-1 PR, D25A mutation resulted in complete inactivation of the enzyme, whereas the T26A mutant showed only residual proteolytic activity. While the mutant HIV-1 PR was virtually monomeric, traces of active dimers were believed to be formed upon interaction with the substrate [23]. In accordance with this, our results imply that PR_PEG10_ has the ability to dimerize; this ability was abolished by the S371A mutation. Results of our in vitro experiments are in good agreement with the predicted structure and prove the essential role of the S371 residue in the dimerization of PR_PEG10_, which depends on “fireman’s grip” interactions, similar to HIV-1 PR.

Previous studies on PEG10′s protease activity implied multiple cleavages within human and mouse PEG10, but the cleavage pattern varied among different cell lines and tissues [12,15,31]. We only observed a single cleavage product (PF1) when anti-PEG10 antibody was used for the detection of proteolytic fragments in HEK293T cells. The molecular weight of PF1 was estimated based on Western blot images and indicated that autoproteolysis may occur prior to the frameshift site, between the capsid-like domain and the Zn-finger motif. The compositions and cavity volumes of the substrate binding sites were determined based on the homology model structure (Figure 3), and the results of in vitro and in silico experiments were used to estimate a potential cleavage position. The highest probability was predicted for the HHQVD*PTEPV cleavage site (Figure 6), which does not resemble most cleavage site sequences of retroviral PRs. Although the presence of an aspartate residue in the P1 position is not characteristic of retroviral PR cleavage sites, it is not unique. For example, the naturally occurring nucleocapsid–protease cleavage site of Walley epidermal hyperplasia virus (WDSV) PR contains a P1-Asp residue (TYPAD*PIDC) [26], and bovine leukemia virus (BLV) PR was also found to efficiently cleave a peptide representing a P1-substituted analogue of the HTLV-1 capsid–nucleocapsid cleavage-site (KTKVD*VVQPK) [32]. Structural analysis of BLV and human T-cell leukemia virus type I (HTLV-1) PRs revealed that both enzymes have mainly hydrophobic S1 binding cavities, which consist of identical residues [25]. In contrast, the P1-Asp-substituted substrate was cleaved efficiently by BLV protease, but not by HTLV-1 PR [25]. This shows that estimation of cleavage positions based purely on the volumes of cleavage site residues and substrate binding cavities should only be considered approximate.

The applied small-scale eukaryotic expression system was found to be not suitable enough to produce the protein in sufficient amounts for cleavage site identification; thus, _fs_RF1/RF2_PEG10_ was expressed in a bacterial expression system. Similar to other retroviral-like proteases—especially recombinant human Ddi2 [27] and *S. cerevisiae* Ddi1 [28] PRs—we also failed to detect the proteolytic activity of the bacterially expressed purified protein. As has already been proposed for Ddi1 and Ddi2 PRs [27,28], putative-specific factors or determinants may be necessary for the activation of the protease, which would probably not be present in the sample after protein purification. Therefore, identification of the autoproteolytic cleavage positions of PEG10 remains to be identified.

Our data imply low catalytic efficiency of PR_PEG10_, but detailed comparison cannot be made due to the limited data on the catalytic efficiencies of retroviral-like proteases. To date, the proteolytic activity of a recombinant protein was proven in vitro only for *Leishmania major* Ddi1 [33] and *Saccharomyces cerevisiae* Ty1 retroviral-like proteases [30], while catalytic efficiency was determined only for the latter one. The structural characteristics of PR_PEG10_ indicate high similarity with other retroviral-like proteases; thus, the dimer stability of PR_PEG10_ is potentially lower than that of most retroviral proteases [31], and is more comparable to that of retroviral-like proteases. For example, Ty1 PR was found to possess trans activity and has very low specific activity as compared to retroviral proteases (e.g., HIV-1, HTLV-1, BLV, and MMLV) [30]. Due to the limited data, future studies need to reveal whether all human retroviral-like proteases (including PEG10, Ddi1, and Ddi2) have lower specific activity than retroviral proteases.

Activity was not detected when a recombinant protein containing putative autoproteolytic sites was used as the substrate; the lack of trans activity indicated a self-inactivation mechanism for PR_PEG10_. The suspected self-inactivation mechanism has already been observed for alphavirus capsid PRs [34]. In these viruses, the nascent structural polyprotein contains an intramolecular (chymotrypsin-like) serine protease that possesses cis activity. As part of polyprotein maturation, self-cleavage occurs, leading to the release of the capsid. This autoproteolytic event is a perquisite for capsid assembly and results in PR inactivation. After self-cleavage, the conserved P1 residue of the cleavage site blocks the trans activity by interacting with the catalytic center [34]. Self-inactivation has also been described for the capsid protease of the Aura virus, Chikungunya virus [35], Sindbis virus [36], and Semliki Forest virus [37], but has not been previously reported in the case of retroviral or retroviral-like PRs. A possible mechanism for blocking trans activity may be caused by a crystal structure of the Ddi1 of *Saccharomyces cerevisiae* [28]. In this structure, the N-terminal region of the homodimeric PR interacts with the active site of the adjacent asymmetric unit as a pseudo-substrate. Although this was considered to be caused by a crystallization artefact, this reveals a possible mode of substrate engagement [28]. We assume that this binding mode may represent a possible mode of inactivation. In this case, the autoproteolytic fragments may interact with the active site of PR_PEG10_, blocking the trans activity. However, future studies are needed to explore whether inter- or intramolecular events are involved in the self-inactivation mechanism.

We detected a difference between the calculated (73 kDa) and observed (~95 kDa) molecular weights of the overexpressed _fs_RF1/RF2_PEG10_ protein (Figure 4, Figure 5, Figure 6, Figure 7, Figure 8, Figure 9 and Figure 10), which implies post-translational protein modification [12,15]. The difference in the molecular weight is attributed to ubiquitination of RF1/RF2_PEG10_, which is supported by the fact that ubiquitination of PEG10 has already been indicated based on its interaction with the E3 ubiquitin-protein ligase SIAH1 protein [4]. Furthermore, quantitative proteomic analyses have reported this post-translational modification [38,39,40,41]. Ubiquitination sites of PEG10 are listed in the PhosphoSite database (http://www.phosphosite.org); our in silico predictions also indicated the presences of putative ubiquitination sites with high confidence (Table S1). Taking into consideration the above-mentioned findings, we assume that the differences in molecular weights of PF1 and PF2 may also be explained by ubiquitination; however, this requires further validation using co-immunoprecipitation techniques in future experiments.

PR_PEG10_ exhibits similarities to other retroviral-like aspartic PRs; however, there are only few studies on the sensitivities of these PRs towards HIV PR inhibitors. Of the tested inhibitors, pepstatin A and saquinavir failed to inhibit the autoproteolytic activity of retroviral-like aspartic protease 1 (ASPRV1), based on literature data [42]. In the case of human Ddi2 protein, isothermal titration calorimetry (ITC) measurements proved that several HIV PR inhibitors, including saquinavir, nelfinavir, darunavir, and acetyl-pepstatin, were unable to bind to the protein, and thus were ineffective against the Ddi2 PR [43]. Recently, human endogenous retrovirus-K (HERV-K) PR was found to be sensitive to darunavir and lopinavir [44]; however, its structure is more similar to that of HIV-1 PR than to those of PEG10, ASPRV1, and Ddi2 PRs. These data suggest that most HIV PR inhibitors have only weak inhibitory potential against retroviral-like human PRs or are unable to inhibit these enzymes. This implies that for an effective inhibition of PR_PEG10_ and its autoproteolytic function, specific inhibitors need to be designed and tested in the future. However, autoproteolysis was not inhibited by the panel of PR inhibitors (Figure 10), we found that self-processing is dependent on the pH (Figure 9), and the optimal pH is close to neutral (6.9–7.4), which is similar to the optimum pH for human foamy virus proteinase (6.6) [45].

To further elucidate the role of PR_PEG10_ in the function of *PEG10*, cell culture experiments were performed, which revealed that PR_PEG10_ plays an important role in the regulation of cell viability and proliferation (Figure 11 and Figure 12). Transfection of cells with _fs_RF1/RF2_PEG10_ harboring wild-type PR_PEG10_ resulted in a significant increase in cellular proliferation compared to cells overexpressing the catalytically inactive protease, at least in the HEK293T cell line. Notably, PR_PEG10_ appeared to decrease cell viability, since transfection of cells with _fs_RF1/RF2_PEG10_ led to a decrease in cell viability by > 60%. This effect was reversed when we inactivated PR_PEG10_ with the D370A mutation. Others have reported that the viability of Alexander and Huh7 cells was also adversely affected by the suppression of endogenous PEG10 expression, unlike in SNU423 cells, which do not express the endogenous protein [4]. The contribution of PEG10 to regulation of cell viability and proliferation has already been studied previously by either overexpressing recombinant PEG10 in transfected cells [4,9,12] or at the level of the endogenous protein (e.g., by downregulating expression by siRNAs) [8,10,11]. While the effects of a recombinant protein on cell viability and proliferation may be different as compared to the endogenous protein, the results obtained by different methods are comparable and reveal the oncogenic potential of PEG10. In agreement with this, in this work we aimed to study the protease activity of PEG10, and we think that the comparison of results obtained for the overexpressed recombinant wild-type and mutant proteins can provide valuable information about protease function.

While the association between *PEG10* and malignancies had already been reported [3,4,5,6,7,8,9,10,11], little is known about the function of PR_PEG10_. We demonstrated that PR_PEG10_ exhibits autoproteolytic activity, and is indeed involved in the cyto-proliferative effect observed when *PEG10* is overexpressed, at least in HEK293T cell line. Although the precise mechanism by which PR_PEG10_ is involved in the induction of cellular proliferation is unclear, we hypothesize that the downregulation of pro-apoptotic pathways mediated by *PEG10* is dependent on a functional protease domain, which may facilitate this interaction in a direct or indirect manner, the details of which should be investigated in the future.

## 4. Materials and Methods

### 4.1. In Silico Analysis

Secondary structure prediction was performed using the Jpred4 server [43] based on the sequence of PR_PEG10_ (UniProtKB: Q86TG7). Disorder prediction was performed using the IUPred web server [46]. Modeller9v13 [47] was used to prepare a homology model for the 346–477 region of RF1/RF2_PEG10_. Crystal structures of EIAV protease (PDBID: 1FMB) [21] and human (PDBID: 3S8I) and yeast (PDBID: 2I1A) Ddi1 proteins [22] were used as templates. Molecular visualizations were performed using the PyMOL Molecular Graphics System (version 1.3; Schrödinger, LLC). The mean cavity volumes of S1–S4 substrate binding sites were calculated for PR_PEG10_ using the previously described method [24,25]. Ubiquitination of human PEG10 was predicted by UbiSite (http://csb.cse.yzu.edu.tw/UbiSite/prediction.php) [48], BDM-PUB: Prediction of Ubiquitination sites with Bayesian Discriminant Method (http://bdmpub.biocuckoo.org), and UbPred (predictor of protein ubiquitination sites) (http://www.ubpred.org/cgi-bin/ubpred/ubpred.cgi) [49] web servers, using default parameters.

### 4.2. Cloning

The PEG10 human cDNA sequence (clone name hh04271) was obtained from Kazusa DNA Research Institute (Kisarazu, Japan). The vector (pQE-TriSystem) for expression of C-terminally 8×His-tagged proteins was a kind gift from Zoltán Papp at the Division of Clinical Physiology, Institute of Cardiology, Faculty of Medicine, University of Debrecen. The cDNA sequences encoding RF1_PEG10_ (1–325 res), RF2_PEG10_ (320–626 res), and RF1/RF2_PEG10_ (1–626 res) were cloned into a HindIII and XhoI enzyme-cleaved pQE-TriSystem expression vector. As the PEG10 cDNA sequence contains an XhoI restriction endonuclease cleavage site, it was mutated to facilitate cloning of the sequence. Insertional mutagenesis was performed in the frameshift site of the sequence encoding RF1/RF2_PEG10_ by insertion of a single adenine into the G-GGA-AAC “slippery” heptanucleotide sequence (G- GGA-AAA-C, inserted nucleotide underlined) to enable transcription of the RF1/RF2_PEG10_-encoding mRNA (_fs_RF1/RF2_PEG10_). A thrombin cleavage site was designed after the PEG10-encoding sequences to facilitate removal of the polyhistidine tag from the fusion protein. The 1878-bp frameshift mutant RF1/RF2_PEG10_, followed by the sequence encoding the thrombin cleavage site, was cloned into the plasmid. The sequences encoding the RF1_PEG10_-thrombin cleavage site, the RF2_PEG10_-thrombin cleavage site, or the RF1/RF2_PEG10_-thrombin cleavage site were amplified in the initial PCR step, while the second PCR reaction was used to create a 3′XhoI restriction site. The inserts were amplified using PCR (iCycler Thermal Cycler, Bio-Rad, Hercules, CA, USA). Both the amplified fragments and the vector were digested with HindIII and XhoI restriction endonucleases and were extracted from agarose gel using a gel extraction kit (ISOLATE II PCR and Gel Kit, BIOLINE) after separation by electrophoresis. For ligation, T4 DNA ligase was used with a vector/insert molar ratio of 1:5. To predict the molecular weight of the C-terminal His-tagged PEG10 proteins, we used a freely available protein molecular weight calculator (http://www.bioinformatics.org/sms/prot_mw.html, date of last accession: June 2017). D369A, D370A, S371A, and XhoI endonuclease cleavage site mutations in the PEG10 sequence were generated using a QuikChange mutagenesis kit (Agilent Technologies, Santa Clara, CA, USA) according to the manufacturer’s instruction. Sequencing was performed using gene- and vector-specific primers and the BigDye Terminator v3.1 Cycle Sequencing Kit (Applied Biosystems, Foster City, CA, USA). Oligonucleotide sequences are shown in Table S2.

### 4.3. Transfection of HEK293T Cells

HEK293T cells (Invitrogen, Carlsbad, CA, USA) were cultured in T-75 flasks in Dulbecco’s modified Eagle’s medium (DMEM) supplemented with 1% penicillin-streptomycin, 1% glutamine, and 10% fetal bovine serum (FBS), and incubated at 37 °C and 5% CO_2_. Cells were transfected with PEG10 constructs (14 μg DNA) at 60% confluency using a polyethylenimine (PEI) transfection protocol [50]. After transfection, the cells were incubated in DMEM containing no antibiotics and FBS. After 5 h of incubation, fresh medium (supplemented with 10% FBS, 1% penicillin-streptomycin, 1% glutamine) was added to cells, and the cells were further incubated overnight. We used pQE-TriSystem plasmid DNA as a mock control.

### 4.4. Lysis of PEG10-Transfected HEK293T Cells

Cells were trypsinized and collected by centrifugation (130× *g*, 8 min, room temperature) 24 h after transfection. The pellet was resuspended in 500 μL of phosphate-buffered saline containing protease inhibitor cocktail (complete EDTA-free Protease Inhibitor Tablet, ROCHE, St. Louis, MO, USA). The pellets were lysed by sonication (Branson Sonifer 450, duty cycle 30 %, output control 4) for 3 × 10 s. Cell lysates were then centrifuged at 16,000× *g* for 15 min at 4 °C to remove cellular debris. For the comparison of expressed PEG10 proteins, 30 μL aliquots of the supernatants were analyzed by Western blot.

### 4.5. Analysis of Proteolytic Activity

Following transfection with either _fs_RF1/RF2_PEG10_ or active-site mutant _fs_RF1/RF2_PEG10_, HEK293T cells were incubated for 48 h and the medium was replaced after 24 h. Next, the cellular pellet was collected and lysed as described above (see “Lysis of PEG10-transfected HEK293T cells”). After cell lysis and centrifugation, supernatants were dialyzed overnight against storage buffer (20 mM piperazine-1,4-bis(2-ethanesulfonic acid) (PIPES), 1 mM EDTA, 100 mM NaCl, 10% glycerol, 0.5% Nonidet P-40, 2 mM DTT, pH 7.0). Protease inhibitor cocktail (20 μL of 7× stock solution) was also added to 120 μL lysate to inhibit the proteolysis of the protein of interest by serine, cysteine, and metalloproteases present in the cellular extract. The proteolytic activity of _fs_RF1/RF2_PEG10_ was assessed by incubating the total cell lysates of transfected cells for 20 h at 37 °C. For the “0 h” sample, the collected cell lysate was frozen immediately at −20 °C.

To investigate the susceptibility of a recombinant protein substrate for cleavage by PR_PEG10_, the total cell lysate of transfected HEK293T cells expressing _fs_RF1/RF2_PEG10_ protein was incubated with His_6_-MBP-_fs_RF1/RF2_PEG10_(1-345)-mTurquoise2 fusion protein in storage buffer for 24 h at 37 °C. The cleavage reaction was followed by Western blot using anti-MBP antiserum (E8030S, New England Biolabs, Ipswich, MA, USA) and anti-PEG10 antibody (SAB1400438-50UG; Sigma-Aldrich, St. Louis, MO, USA).

### 4.6. Recombinant Protein Substrate Preparation

For preparation of a recombinant fusion protein substrate containing the 1–345 residues of the full-length _fs_RF1/RF2_PEG10_ protein (His_6_-MBP-_fs_RF1/RF2_PEG10_ (1-345)-mTurquoise2), we used a slightly modified version of the previously described method [51]. The introduced changes are detailed below.

A pGEX-4T-3 expression plasmid containing the codon-optimized coding sequence of the _fs_RF1/RF2_PEG10_ protein was obtained from GenScript, and the pDest-His_6_-MBP-mTurquoise2 bacterial expression plasmid was used from the in-house stock [51] The coding sequence of the 1–345 region of the _fs_RF1/RF2_PEG10_ protein was amplified using PCR, and both the amplicon and pDest-His_6_-MBP-mTurquoise2 plasmid were digested with PacI and NheI restriction endonucleases followed by ligation. The plasmid was transformed into BL21(DE3) *Escherichia coli* cells, and then the His_6_-MBP-_fs_RF1/RF2_PEG10_(1–345)-mTurquoise2 protein substrate was expressed at 16 °C for 6 h. After cell lysis, the protein substrate was purified by Ni-NTA magnetic agarose beads (Qiagen, Hilden, Germany), after which the buffer was changed to modified storage buffer (20 mM PIPES, 100 mM NaCl, 0.05% Tween20, pH 7.0) using 0.5 mL 10K Amicon Ultra centrifugal filter tubes (Merck-Millipore, Burlington, MA, USA). The purified recombinant protein was applied as the substrate to test the proteolytic activity of PR_PEG10_.

### 4.7. Western Blot Analysis

Samples were prepared for Western blot as described in the Section 4.5. Protein samples subjected for the detection of ubiquitination were purified with Ni-chelate affinity chromatography (HisTrap HP column, GE Healthcare; ÄKTA prime liquid chromatograph, Amersham Biosciences, Little Chalfont, UK). The SDS loading dye was added to the samples and the mixtures were boiled at 95 °C for 10 min, followed by brief centrifugation. Samples were then loaded onto a 14% SDS polyacrylamide gel. Following SDS-PAGE electrophoresis, proteins were transferred onto a nitrocellulose membrane at 100 V for 70 min. The membrane was blocked by 5 % dry milk in Tris-buffered saline (TBS, pH 7.5) for 1 h at room temperature, followed by incubation with the antibodies. Mouse anti-PEG10 polyclonal antibody (SAB1400438-50UG; Sigma-Aldrich, St. Louis, MO, USA) was applied in a 1:2000 dilution, a rabbit anti-ubiquitin polyclonal antibody (Z0458; Dako Cytomation, Glostrup, Denmark) in a 1:500 dilution, and a rabbit anti-MBP antiserum (E8030S, New England Biolabs, Ipswich, Massachusetts, USA) in a 1:20,000 dilution. Rabbit anti-GAPDH antibody (G9545; Sigma-Aldrich, MO, USA) was applied in a 1:15,000 dilution. Primary antibodies were diluted in Tris-buffered saline complemented with Tween20 (TTBS) containing 0.1 % dry milk and were incubated at 4 °C overnight. The membranes were washed three times with TTBS for 15 min and were then incubated with polyclonal anti-mouse (A4416; Sigma-Aldrich, MO, USA) or anti-rabbit (170–6515; Bio-Rad, CA, USA) secondary antibodies for 1 h at room temperature. The membranes were subsequently washed a further three times with TTBS. Proteins were detected using SuperSignal West Pico chemiluminescent substrate (Thermo Fisher Scientific, Waltham, MA, USA).

### 4.8. Determination of pH Optimum for PR_PEG10_

Effect of pH on PR_PEG10_ activity was measured in META buffer (100 mM 2-(N-morpholino)-ethanesulfonic acid (MES), 200 mM Tris, 100 mM Na-acetate, 2 M NaCl). Following transfection of cells, 100 μL of cell lysate was dialyzed against storage buffer (pH 7.0) for 3 h and then incubated at 37 °C for 20 h in a 1:1 volume ratio of a series of META buffers with pH values ranging from 5.0 to 8.0. The pH values of the reactions mixtures were determined to be 5.4, 5.7, 6.0, 6.5, 6.9, 7.4, and 7.9.

### 4.9. In Vitro Inhibition of PR_PEG10_

Pepstatin A, lopinavir, nelfinavir, saquinavir, darunavir, and tipranavir (NIH AIDS Reagent Program) dissolved in dimethylsulfoxide (DMSO, Sigma-Aldrich, MO, USA) were used for the inhibition of PR_PEG10_. The reaction mixtures contained the lysate of _fs_RF1/RF2_PEG10_-transfected cells, which was dialyzed against storage buffer. HIV inhibitors were added to a final concentration of 10 µM, followed by incubation at 37 °C for 20 h. Only DMSO was added to the control sample. The reactions were terminated by the addition of SDS loading dye, and the mixtures were boiled at 95 °C for 10 min and centrifuged briefly before Western blot analysis.

### 4.10. Cell Viability Assay

HEK293T and HaCaT cells were seeded at a density of 10,000 cells per well (~70–80% confluence) in 96-well plates 16 h before treatment. For transfection of the cells, Lipofectamine LTX&PLUS™ reagent (Invitrogen, Carlsbad, CA, USA) was used according to the manufacturer’s instructions. The plasmid DNA (100 ng/well) and the Lipofectamine reagent (0.5 μL/well) were also diluted in Opti-MEM medium. After incubation for 5 min, the DNA was mixed with the diluted Lipofectamine in a 1:1 volume ratio. The DNA–lipid mixture was incubated for 20 min at room temperature. The cell culture medium was changed to fresh Opti-MEM (90 μL) in each well, and after addition of the pre-incubated mixture the cells were further incubated for 5 h, then the transfection medium was replaced with fresh DMEM. Cell viability was determined by adding 1 mM of 3-(4,5-dimethylthiazol-2-yl)-2,5-diphenyltetrazolium bromide (MTT, Invitrogen, Carlsbad, CA, USA) to the cells that were incubated at 37 °C for 4 h. Then, MTT formazan crystals were dissolved using DMSO (Sigma-Aldrich, MO, USA), followed by re-incubation for 10 min. Absorbance values were measured at 544 nm using an automatic microplate reader (Wallac 1420 Victor2, Wallac Oy, Turku, Finland). HEK293T cells transfected with a pQE-TriSystem plasmid were used as mock control, whereas the efficiency of transfection was determined by measuring fluorescence of cells transfected with a pQE-TriSystem-GFP plasmid.

### 4.11. Analysis of Cell Proliferation by Flow Cytometry

HEK293T and HaCaT cells maintained in DMEM (supplemented with 1% penicillin-streptomycin, 1% glutamine, and 10% FBS) were plated in 6-well plates at a density of 3 × 10^5^ cells/well 24 h before transfection. Cells maintained in Opti-MEM medium lacking antibiotics were transfected with _fs_RF1/RF2_PEG10_ or D370A-_fs_RF1/RF2_PEG10_ DNA, using Lipofectamine LTX&PLUS™ reagent (Invitrogen, CA, USA) according to the manufacturer’s protocol. Then, 5 μL/well Lipofectamine reagent and 3 μg/well plasmid DNA were diluted in Opti-MEM medium and incubated for 5 min. After incubation, the DNA and the Lipofectamine were mixed (volume ratio of 1:1). In order to form the DNA-lipid complex, the solution was incubated again for 20 min. The incubated solution was added to the cells in 750 μL Opti-MEM dropwise and incubated for 5 h. For mock control, pQE-TriSystem was used to transfect cells. After incubation, 3 mL of fresh medium supplemented with 10% FBS was added to the transfected cells, which were then further incubated for 36 h. The success of transfection was verified by determining the fluorescence of control cells that had been transfected with pQE-TriSystem-GFP plasmid. Cellular proliferation was analyzed using flow cytometry (FACS Calibur, BD Biosciences, San Jose, CA, USA), using mouse-anti-human Ki67-FITC antibody (11-5699-41, eBioscience, San Diego, CA, USA).

### 4.12. Statistical Analysis

The GraphPadQuickCalcs unpaired *t*-test free web calculator was used for statistical analysis (http://graphpad.com/quickcalcs/ttest2) (accessed June 2019).

## Supplementary Materials

Supplementary materials can be found at https://www.mdpi.com/1422-0067/21/7/2424/s1.

## Abbreviations

**ASPRV1**	Retroviral-like aspartic protease 1
**Ddi1**	DNA damage-inducible protein 1
**Ddi2**	DNA damage-inducible protein 2
**DMEM**	Dulbecco’s modified Eagle’s medium
**DMSO**	Dimethylsulfoxide
**EIAV**	Equine infectious anemia virus
**HaCaT**	Human keratinocyte cells
**HEK293T**	Human embryonic kidney cells
**HIV-1**	Human immunodeficiency virus type 1
**MTT**	3-(4,5-dimethylthiazol-2-yl)-2,5-diphenyltetrazolium bromide
**PEG10**	Paternally expressed gene 10
**PF1**	Processing fragment 1
**PF2**	Processing fragment 2
**PR**	Protease
**PR_PEG10_**	Retroviral-like protease domain in RF1/RF2_PEG10_ protein
**RF1_PEG10_**	*Gag*-like protein encoded by PEG10
**RF2_PEG10_**	*Pol*-like protein encoded by PEG10
**RF1/RF2_PEG10_**	*Gag-Pol*-like protein encoded by PEG10
**_fs_RF1/RF2_PEG10_**	Frameshift mutant *Gag-Pol*-like protein encoded by PEG10
**TEV**	Tobacco etch virus
**TBS**	Tris-buffered saline
**TTBS**	Tris-buffered saline complemented with Tween20
