# Supplementary material for: Functional Study of the Retrotransposon-Derived Human PEG10 Protease
Source: Int J Mol Sci. 2020 Mar 31;21(7):2424. doi: 10.3390/ijms21072424 (PMC7212762; doi:10.3390/ijms21072424)
Supplement: Supplementary file 1 [file ijms-21-02424-s001.zip › Table S1_IJMS_PEG10.docx]

**Table S1: Ubiquitination of PEG10.** The sequences harbouring the ubiquitination sites are shown, ubiquitinated residues are bold and underlined. Experimentally determined sites are shown based on PhosphoSitePlus database ([www.phosphosite.org](http://www.phosphosite.org)) (accessed at 2019.10.22). LTP: Number of records, modification determined by methods other that discovery mass spectrometry, HTP: Number of records, modification determined by only proteomic discovery mass spectrometry. Predicted scores are shown for all residues, and the confidence is also indicated if it was determined by the web server. For BDM-PUB prediction, default threshold was 0.3; “-“ indicates that no ubiquitination was predicted for the given site.

|  |  | **PhosphoSitePlus** | | **UbiSite** | **BDM-PUB** | **UbPred** |
| --- | --- | --- | --- | --- | --- | --- |
| **Site** | **Sequence** | LTP | HTP | value / confidence | value | value / confidence |
| K19 | INNLRE**K**VMKQSE | 0 | 0 | 0.311656 (low) | 0.89 | 0.72 (medium) |
| K22 | LREKVM**K**QSEENN | 0 | 21 | 0.318768 (low) | - | 0.76 (medium) |
| K36 | LQSQVQ**K**LTEENT | 0 | 10 | 0.526757 (high) | - | 0.86 (high) |
| K85 | PEDLPE**K**FDGNPD | 0 | 0 | 0.553221(high) | - | 0.87 (high) |
| K136 | ARWASA**K**LERSHY | 0 | 28 | 0.487195 (medium) | 1.87 | - |
| K155 | AFMMEM**K**HVFEDP | 0 | 0 | 0.3167 (low) | - | - |
| K168 | QRREVA**K**RKIRRL | 0 | 0 | - | 1.65 | - |
| K170 | REVAKR**K**IRRLRQ | 0 | 0 | - | 1.87 | - |
| K225 | SHLEVA**K**SLSALI | 0 | 0 | 0.380662 (medium) | 1.45 | - |
| K249 | RAAAAR**K**PRSPPR | 0 | 0 | - | 3.50 | - |
| K286 | RLTQEE**K**ERRRKL | 0 | 1 | 0.438893 (medium) | - | - |
| K291 | EKERRR**K**LNLCLY | 0 | 22 | 0.290411 (low) | 0.49 | - |
| K311 | ADNCPA**K**ASKSSP | 0 | 0 | - | 2.21 | 0.65 (low) |
| K314 | CPAKAS**K**SSPAGK | 0 | 0 | 0.301204 (low) | 3.80 | 0.68 (low) |
| K320 | KSSPAG**K**LPGPAV | 0 | 0 | 0.494103 (medium) | 3.08 | 0.74 (medium) |
| K393 | GIPLRI**K**DWPILV | 0 | 0 | 0.604822 (high) | - | - |
| K567 | FVARNV**K**DGLITP | 0 | 62 | 0.44881 (medium) | - | - |
| K586 | AQVLQV**K**RGWKLQ | 0 | 49 | 0.393159 (medium) | 0.94 | - |
| K590 | QVkRGW**K**LQVSYD | 0 | 27 | 0.453299 (medium) | 1.94 | - |
