# Supplementary material for: Functional Study of the Retrotransposon-Derived Human PEG10 Protease
Source: Int J Mol Sci. 2020 Mar 31;21(7):2424. doi: 10.3390/ijms21072424 (PMC7212762; doi:10.3390/ijms21072424)
Supplement: Supplementary file 1 [file ijms-21-02424-s001.zip › Table S2_IJMS_PEG10.docx]

**Table S2. Primers used for cloning and mutagenesis.** The listed primer sequences are available in the public oligonucleotide database of the Laboratory of Retroviral Biochemistry (<http://lrb.med.unideb.hu/index.php/research/oligos>).

| **Primer number** | **Primer name** | **Sequence** |
| --- | --- | --- |
| **1.** | PEG10 XhoI mutagenesis | 5′-gagctctcccacctggaggtcgccaagtcg-3′ |
| **2.** | PEG10 frameshift mutant primer | 5′-cttcgccggcgggaaaactccccggcccc-3′ |
| **3.** | RF1_PEG10_ and RF1/RF2_PEG10_ pQE-TriSystem forward- HindIII | 5′-gctcaagcttatgaccgaacgaagaagggac-3′ |
| **4.** | RF2_PEG10_ pQE-TriSystem forward- HindIII | 5’-gctcaagcttaaactccccggccccgctg-3′ |
| **5.** | RF1_PEG10_ pQE-TriSystem reverse1-Thrombin | 5′-ggatccacgcggaaccagcagcggggccggggagtttc-3′ |
| **6.** | RF2_PEG10_ and RF1/RF2_PEG10_ pQE-TriSystem reverse1- Thrombin | 5′-ggatccacgcggaaccaggtacgttgccaggtgtgc-3′ |
| **7.** | RF1_PEG10_, RF2_PEG10_ and RF1/RF2_PEG10_ pQE-TriSystem reverse2-XhoI | 5′-ctactcgagggatccacgcgaaccag-3′ |
| **8.a** | PR_PEG10__D369A/D370A forward primer | 5′-ccgaggccatgatcgcttctggtg-3′ |
| **8.b** | PR_PEG10__D369A/D370A reverse primer | 5′-ccagaagcaccagaagcgatcatggctcgg-3′ |
| **9.a** | PR_PEG10__S371A forward primer | 5′-gagccatgatcgatgctggtgcttctggc-3′ |
| **9.b** | PR_PEG10__S371A reverse primer | 5′-gccagaagcaccagcatcgatcatggctc-3′ |
| **10.a** | His_6_-MBP-_fs_RF1/RF2_PEG10_-mTurquoise2 forward primer | 5′-gcttaattaaaatgaccgagcgtcgtcgtg-3′ |
| **10.b** | His_6_-MBP-_fs_RF1/RF2_PEG10_-mTurquoise2 reverse primer | 5′-tagctagcgctcgcatcatcttgcggg-3′ |
